# Association between the number of existing teeth and maintenance dialysis therapy: A cross-sectional study of adult male dentists

**DOI:** 10.1371/journal.pone.0309012

**Published:** 2024-08-16

**Authors:** Minami Kondo, Marin Ishigami, Maho Omoda, Moeno Takeshita, Nishiki Arimoto, Rumi Nishimura, Tomoko Maehara, Toru Naito, Masaaki Kojima, Osami Umemura, Makoto Yokota, Nobuhiro Hanada, Kenji Wakai, Mariko Naito

**Affiliations:** 1 R&D, SUNSTAR Inc., Takatsuki, Osaka, Japan; 2 Midori Health Center, Nagoya, Aichi, Japan; 3 Aoba Ward Welfare and Health Center, Yokohama City, Kanagawa, Japan; 4 Department of Oral Health Sciences, Otemae College, Nishinomiya, Hyogo, Japan; 5 Department of Oral Epidemiology, Graduate School of Biomedical and Health Sciences, Hiroshima University, Hiroshima, Japan; 6 Department of Public Oral Health, Graduate School of Biomedical and Health Sciences, Hiroshima University, Hiroshima, Japan; 7 Department of General Dentistry, Fukuoka Dental College, Fukuoka, Japan; 8 Aichi Dental Association, Nagoya, Aichi, Japan; 9 Yokota Makoto Dental Clinic, Fukuoka, Japan; 10 Institute of Photochemistry and Photocatalyst, University of Shanghai for Science and Technology, Shanghai, China; 11 Department of Preventive Medicine, Nagoya University Graduate School of Medicine, Nagoya, Aichi, Japan; University of Catania: Universita degli Studi di Catania, ITALY

## Abstract

Dental caries and periodontal disease are typical oral diseases frequently observed in patients with renal diseases. Tooth loss is an outcome of dental caries and periodontal disease, and the number of existing teeth is an indicator of oral health status. However, the association between the number of existing teeth and end-stage kidney disease (ESKD) has not been investigated in detail. This study aimed to investigate the association between oral health status, expressed by the number of existing teeth, and ESKD. We analyzed data from the second survey of the Longitudinal Evaluation of Multi-phasic, Odontological, and Nutritional Associations in Dentists, a cohort study conducted among members of the Japan Dental Association. From August 2016 to July 2017, self-administered questionnaires were mailed to 16,128 male dentists and 8,722 responded. Among them, 7,479 men with complete data on age, number of existing teeth, and ESKD were included in the analysis. Multivariate logistic regression analysis was conducted, with ESKD as the dependent variable and the number of existing teeth (≥23 teeth and <23 teeth) as the independent variable. Subgroup analysis by age (<65 years and ≥65 years) was also conducted. The <23 teeth group had a significantly higher rate of ESKD than did the ≥23 teeth group. After adjusting for age, body mass index, smoking habits, hypertension, and diabetes mellitus, there was no significant association between having <23 teeth and ESKD in all participants. However, the subgroup analysis revealed a significant association after adjustment for covariates in participants aged <65 years but not in those aged ≥65 years. In conclusion, having <23 teeth was associated with the risk of requiring maintenance dialysis therapy among Japanese men aged <65 years. Therefore, tooth loss may be associated with renal function decline.

## Introduction

The incidence of chronic kidney disease (CKD) is increasing worldwide [1]. CKD is projected to become the fifth most prevalent non-communicable disease globally by 2040 [2]. Renal replacement therapy, such as maintenance dialysis therapy, is necessary when CKD progresses to end-stage kidney disease (ESKD). As of 2022, the number of patients undergoing maintenance dialysis therapy in Japan was approximately 1 in 350 [3], which was the third-highest rate worldwide [4]. A previous study reported an independent graded association of the CKD stage with the risk of death, cardiovascular disease, and hospitalization [5]. Furthermore, men progress more rapidly to CKD than women [6, 7], and the risk of progression to ESKD is 50% higher in men [8]. Therefore, the progression of CKD and the development of ESKD are important clinical and public health issues because they increase the risk of adverse outcomes, and their effects may be greater in men.

The association between poor oral health and renal function decline has recently received increasing attention. Dental caries and periodontal disease are among the most prevalent diseases globally [9] and are common in patients with renal disease. Patients undergoing hemodialysis (HD) have more untreated and severe dental caries [10, 11] and a higher incidence and severity of periodontal disease [12]. Furthermore, previous studies have suggested that dental caries and periodontal disease may affect the survival of patients with ESKD [13, 14]. Dental caries and periodontal disease are both caused by dental plaque. Dental caries is the demineralization and decay of teeth [15], while periodontal disease is a chronic inflammatory disease [16]. Both conditions can be corrected by lifestyle modifications and dental treatment, which may be useful in controlling risk factors for renal function decline. Therefore, it is important to elucidate the association between poor oral health and renal function decline.

Dental caries and periodontal disease are the major causes of tooth loss [17], and the number of existing teeth is an indicator of oral health status. Nevertheless, to our knowledge, only a few studies have investigated the association between the number of existing teeth and ESKD. Additionally, both tooth loss and ESKD are influenced by socioeconomic status, which may confound their association [18, 19]. This study aimed to investigate the association between the number of existing teeth and ESKD among Japanese male dentists, a group with relatively homogeneous socioeconomic status.

## Materials and methods

### Study design and participants

This cross-sectional study analyzed the second survey data from the Longitudinal Evaluation of Multi-phasic, Odontological, and Nutritional Associations in Dentists (LEMONADE) cohort study. Participants in the LEMONADE study were members of 46 prefectural dental associations affiliated with the Japan Dental Association (JDA). The JDA is a unique dental professional organization that primarily comprises dental practitioners, enrolling 67.2% of all dentists in Japan as of the end of 2006. A baseline survey [20] was conducted between February 2001 and July 2006 among dentists affiliated with 1 of 46 prefectural dental associations. The questionnaire was delivered to 58,792 JDA members. In total, 21,272 dentists were registered in the baseline survey. The recruitment of participants for the second survey was conducted from August 1, 2016 to March 31, 2017 among 40 prefectural dental associations that participated in the baseline survey. Self-administered questionnaires were mailed to 16,128 participants.

The self-administered questionnaire contained questions regarding medical history, oral health, smoking habits, and systemic conditions. The participants were asked about the number of missing teeth. The number of existing teeth was then calculated by subtracting the number of missing teeth from 28. Maintenance dialysis therapy was used as an indicator of ESKD. ESKD was defined according to the response to the question, “Do you have regular hemodialysis or peritoneal dialysis (more than 1 month since the start of treatment)?”. Those who answered “Yes” were asked about the primary reasons for maintenance dialysis therapy. The options were diabetes mellitus, chronic nephritis, nephrosclerosis, and others. The participants were asked if they had ever been diagnosed with hypertension or diabetes mellitus. The use of medications for hypertension and diabetes mellitus (oral antidiabetic agents or self-injected insulin) was also self-reported. Hypertension was defined according to its diagnosis or use of medications for hypertension. Diabetes mellitus was defined according to its diagnosis, administration of oral antidiabetic agents, or administration of self-injected insulin. Smoking habit was defined according to the response to the question, “Have you ever smoked almost daily for at least 1 year?”. Participants who answered “No” were classified into the never-smoker group, those who answered “I used to smoke” were classified into the former-smoker group, and those who answered “I currently smoke” were classified into the current-smoker group.

This study was approved by the Ethics Committee of Nagoya University School of Medicine (approval number 2008–0632) and the Ethical Committee for Epidemiology of Hiroshima University (approval number 2019–1603). This study was conducted according to the principles expressed in the Declaration of Helsinki and in line with the STROBE guidelines for cross-sectional studies. All participants provided written informed consent before the procedure commenced.

### Statistical analysis

Participants were categorized into two groups (≥23 teeth and <23 teeth) based on the mean number of existing teeth in men aged 60–64 years in the 2016 Survey of Dental Diseases in Japan [21]. Body mass index (BMI) was calculated by dividing self-reported body weight (kg) by the height squared (m^2^). BMI was categorized following the Japan Society for the Study of Obesity classifications as underweight, BMI <18.5 kg/m^2^; normal, BMI 18.5 kg/m^2^ to <25 kg/m^2^; and obese, BMI ≥25 kg/m^2^.

The characteristics of the participants according to the number of existing teeth were analyzed using chi-squared tests and were presented as frequency distributions of categorical variables. The adjusted odds ratio (aOR) and 95% confidence interval (CI) for the association between ESKD (dependent variable) and the number of existing teeth (independent variable) were calculated using a logistic regression model. Model 1 was adjusted for age. Model 2 included the confounders from Model 1, as well as BMI and smoking habits. Model 3 was adjusted for the confounders in Model 2, plus hypertension and diabetes mellitus. Additionally, subgroup analyses by age were conducted. Based on the general definition of elderly and a previous study [22], a cutoff age of 65 years was used. All statistical analyses were conducted using the SPSS statistical software version 28.0 (IBM Co., Armonk, NY, USA). P*-*values <0.05 were considered statistically significant.

## Results

Of the 16,128 participants, 8,722 participants completed and returned the questionnaire to the central office. Among them, 1,243 were excluded because they were women (n = 600) and had missing data on age, number of existing teeth, and ESKD (n = 643). Therefore, 7,479 men (aged 39–100 years) were included in the analysis. The mean age was 62±9 years, and 32 patients (0.4%) underwent maintenance dialysis therapy. The characteristics of the participants according to the number of existing teeth are presented in Table 1. Compared with the ≥23 teeth group, the <23 teeth group had significantly higher rates of older age, underweight, hypertension, diabetes mellitus, former smokers, current smokers, and ESKD. However, the primary reason for maintenance dialysis therapy was not significantly different between the two groups.

**Table 1:** Characteristics of the participants according to the number of existing teeth.

Variables	≥23 teeth	<23 teeth	P-value^†^
**Age (years)**			<0.001^‡^
≤49	504 (8.0)	19 (1.6)	
50–59	2,406 (38.4)	150 (12.3)	
60–69	2,489 (39.7)	458 (37.7)	
70–79	712 (11.4)	343 (28.2)	
80–89	146 (2.3)	216 (17.8)	
≥90	6 (0.1)	30 (2.5)	
**BMI (kg/m** ^**2**^ **)** *			<0.001^‡^
Underweight	152 (2.4)	52 (4.3)	
Normal	4,367 (70.0)	847 (70.3)	
Obese	1,716 (27.5)	306 (25.4)	
**Hypertension**			<0.001^‡^
No	4,072 (65.2)	624 (51.4)	
Yes	2,177 (34.8)	590 (48.6)	
**Diabetes mellitus**			<0.001^‡^
No	5,533 (88.5)	970 (79.9)	
Yes	717 (11.5)	244 (20.1)	
**Smoking habits**			0.028^‡^
Never smoker	5,108 (81.7)	954 (78.6)	
Former smoker	198 (3.2)	51 (4.2)	
Current smoker	946 (15.1)	208 (17.1)	
**Maintenance dialysis therapy**			0.021^‡^
No	6,241 (99.6)	1,206 (99.2)	
Yes	22 (0.4)	10 (0.8)	
**Primary reason for maintenance dialysis therapy**			0.915
Diabetes mellitus	6 (33.3)	3 (37.5)	
Chronic nephritis	5 (27.8)	2 (25.0)	
Nephrosclerosis	1 (5.6)	1 (12.5)	
Others	6 (33.3)	2 (25.0)	

Values are presented as numbers (%).

*BMI, body mass index; underweight, BMI <18.5 kg/m^2^; normal, BMI 18.5 kg/m^2^ to <25 kg/m^2^; obese, BMI ≥25 kg/m^2^.

^†^Obtained from chi-square test.

^‡^Statistical significance at P <0.05.

Table 2 presents the results of the multiple logistic regression analysis of the association between the number of existing teeth and ESKD in all participants. There was no significant association between having <23 teeth and ESKD after adjustment for age, BMI, smoking habits, hypertension, and diabetes mellitus in all participants (aOR = 1.5, 95%CI: 0.6–3.5, P = 0.350).

**Table 2:** Multiple logistic regression analysis of the association between the number of existing teeth and ESKD in all participants.

		n	Maintenance dialysis therapy	Coefficient	SE	aOR (95%CI)	P-value^§^
Model 1*	≥23 teeth	6,263	22			1 (reference)	
	<23 teeth	1,216	10	0.507	0.431	1.7 (0.7–3.9)	0.239
Model 2^†^	≥23 teeth	6,224	22			1 (reference)	
	<23 teeth	1,202	10	0.523	0.438	1.7 (0.7–4.0)	0.232
Model 3^‡^	≥23 teeth	6,209	22			1 (reference)	
	<23 teeth	1,200	10	0.405	0.434	1.5 (0.6–3.5)	0.350

SE, standard error; aOR, adjusted odds ratio; CI, confidence interval.

*Model 1 was adjusted for age.

^†^Model 2 was adjusted for age, BMI, smoking habits.

^‡^Model 3 was adjusted for age, BMI, smoking habits, hypertension, and diabetes mellitus.

^§^Statistical significance at P <0.05.

Hosmer–Lemeshow goodness of fit test: Model 1, P = 0.695; Model 2, P = 0.953; Model 3, P = 0.315.

Table 3 shows the results of the multiple logistic regression analysis of the association between the number of existing teeth and ESKD in participants categorized by age. In participants aged <65 years, there was a significant association between having <23 teeth and ESKD after adjustment for age, BMI, smoking habits, hypertension, and diabetes mellitus (aOR = 4.4, 95%CI: 1.3–15.3, P = 0.019). In contrast, there was no significant association between having <23 teeth and ESKD in participants aged ≥65 years (aOR = 0.9, 95%CI: 0.3–2.6, P = 0.835).

**Table 3:** Multiple logistic regression analysis of the association between the number of existing teeth and ESKD in participants categorized by age.

			n	Maintenance dialysis therapy	Coefficient	SE	aOR (95%CI)	P-value^§^
<65 years	Model 1*	≥23 teeth	4,308	9			1 (reference)	
		<23 teeth	359	4	1.579	0.616	4.9 (1.5–16.2)	0.010
	Model 2^†^	≥23 teeth	4,280	9			1 (reference)	
		<23 teeth	354	4	1.600	0.622	5.0 (1.5–16.8)	0.010
	Model 3^‡^	≥23 teeth	4,270	9			1 (reference)	
		<23 teeth	354	4	1.485	0.635	4.4 (1.3–15.3)	0.019
≥65 years	Model 1*	≥23 teeth	1,955	13			1 (reference)	
		<23 teeth	857	6	−0.005	0.527	1.0 (0.4–2.8)	0.993
	Model 2^†^	≥23 teeth	1,944	13			1 (reference)	
		<23 teeth	848	6	0.014	0.539	1.0 (0.4–3.0)	0.979
	Model 3^‡^	≥23 teeth	1,939	13			1 (reference)	
		<23 teeth	846	6	−0.113	0.543	0.9 (0.3–2.6)	0.835

SE, standard error; aOR, adjusted odds ratio; CI, confidence interval.

*Model 1 was adjusted for age.

^†^Model 2 was adjusted for age, BMI, and smoking habits.

^‡^Model 3 was adjusted for age, BMI, smoking habits, hypertension, and diabetes mellitus.

^§^Statistical significance at P <0.05.

Hosmer–Lemeshow goodness of fit test: Age <65 years: Model 1, P = 0.916; Model 2, P = 0.904; Model 3, P = 0.694; Age ≥65 years: Model 1, P = 0.690; Model 2, P = 0.940; Model 3, P = 0.492.

## Discussion

This cross-sectional study evaluated the association between the number of existing teeth and ESKD in Japanese men. After adjusting for age, BMI, smoking habits, hypertension, and diabetes mellitus, having <23 teeth was significantly associated with ESKD in participants aged <65 years. A study of Americans aged 25–64 years reported a significantly higher percentage of renal disease in those with ≥6 missing teeth than in those without tooth loss or with 1–5 missing teeth [23]. Our results support those of the previous research.

The following mechanisms may explain the association between the number of existing teeth and ESKD. In a study that systematically evaluated the prevalence of oral disease in patients with renal disease, xerostomia occurred in approximately half of patients undergoing maintenance dialysis therapy [24]. Although the mechanism underlying the development of xerostomia in patients undergoing maintenance dialysis therapy is unknown, it is thought to be due to fluid restriction or side effects of drug therapy [25]. Individuals with xerostomia have a higher risk of dental caries than those without xerostomia [26]. A previous study evaluating the dental caries status in patients undergoing HD found that untreated caries and the rate of xerostomia were significantly higher in the patients, suggesting that xerostomia may play a role in the development of dental caries in patients undergoing maintenance dialysis therapy [10]. Therefore, xerostomia in patients undergoing maintenance dialysis therapy may promote the development and progression of dental caries, leading to tooth loss.

Periodontal disease is another cause of tooth loss. Most causative organisms of periodontal disease are gram-negative bacteria [27]. The outer cell wall membrane of gram-negative bacteria comprises lipopolysaccharide, an endotoxin. Compared with healthy individuals, patients with periodontal disease have significantly higher blood levels of inflammatory cytokines, which are derived from the endotoxins of gram-negative bacteria, and C-reactive protein (CRP) [28, 29]. CRP is a known systemic inflammatory marker induced in the liver cells by inflammatory cytokines. These findings suggest that inflammatory cytokines produced in periodontal pockets may contribute to the systemic inflammatory burden via the blood. A previous study found a significant positive correlation between periodontal clinical parameters and blood CRP levels in patients undergoing HD [30]. Furthermore, elevated blood levels of inflammatory cytokines and CRP have been reported as predictors of renal function decline [31]. Therefore, periodontal disease may mediate immune responses in patients with renal disease, and tooth loss resulting from the progression of periodontal disease may be involved in the deterioration of renal function and ESKD.

Another reason for the observed association between periodontal disease and ESKD may be genetic factors. Associations between various genetic polymorphisms and renal function decline have been reported in recent years. A study of Korean patients undergoing HD reported that the carriage rate of the interleukin-6 (IL-6) -634 G allele was significantly higher in patients undergoing HD than in healthy individuals [32]. In a study of White patients with chronic glomerulonephritis, those with faster progression to ESKD had significantly higher carriage rates of IL-6–634 G allele than those with slower progression. Multivariate logistic regression analysis showed a significant association between the IL-6–634 G allele and ESKD progression [33]. IL-6–634 G allele promotes IL-6 production and secretion [34]. IL-6 in turn promotes osteoclast function and plays an essential role in bone resorption [35], suggesting that IL-6 is involved in the loss of alveolar bone, which supports the teeth. In a study on Japanese people, even after adjusting for confounding factors, those with the GG genotype of IL-6–634 had significantly fewer teeth than those with the CC genotype, and the number of existing teeth decreased as the number of G alleles increased [36].

This study found a significant association between having <23 teeth and ESKD in participants aged <65 years. A previous study showed that the percentage of patients undergoing maintenance dialysis therapy who reported a family history of ESKD was significantly higher among middle-aged patients than older patients, suggesting that genetic factors may be involved in the development of ESKD at a younger age [37]. Therefore, genetic factors may accelerate the development of ESKD in middle-aged patients, and inflammatory cytokine gene polymorphisms associated with the progression to ESKD may also affect tooth loss. Further studies that include genetic factors are necessary.

The strength of our study is that a higher validity can be expected in self-reports of the number of existing teeth because they are dentists. Previous studies have suggested that self-reporting the number of existing teeth is a valid alternative to clinical examinations in the general population [38]. Dentists are even more likely to report accurate oral health data because of their extensive oral health knowledge. Additionally, the socioeconomic characteristics of the study population, such as education and income, are expected to be relatively homogeneous; hence, confounding based on socioeconomic status or access to medical services is unlikely in this study.

However, this study also had some limitations. First, the generalizability of the results is limited. The participants in this study were exclusively male dentists affiliated with the JDA. Therefore, the findings may differ from those of more heterogeneous populations, such as women and individuals from different occupational or socioeconomic backgrounds. Second, confounding could not be entirely eliminated. Although this study adjusted for specific confounders such as age, BMI, smoking habits, hypertension, and diabetes mellitus, other factors may influence the association between the number of existing teeth and ESKD. For example, a previous study [39] reported that a plant-based diet is a protective factor for renal function decline. Additionally, alcohol consumption of 60 g or more per day has been reported to be a risk factor for renal function decline in men [40]. However, information on dietary intake and alcohol consumption habits was not available and could not be accounted for in this study. Future research should include these factors to provide a more comprehensive understanding. Third, the cross-sectional design of this study limits its ability to establish a causal relationship. The observed association between tooth loss and ESKD does not imply causation. Longitudinal studies are needed to clarify the temporal sequence of events and determine the causal relationship more definitively. Fourth, recall bias may affect the results, as the study relies on self-reported data for all variables. Self-reported data are generally susceptible to recall bias, which means the data might not accurately reflect the true status of the participants. Fifth, the study failed to consider other oral health factors beyond the number of existing teeth. While this study focused solely on the number of existing teeth as an indicator of oral health status, other factors such as caries experience, periodontal status, oral hygiene practices, and oral health behaviors may also influence renal function decline. A more comprehensive assessment of oral health status could provide a clearer understanding of its association with renal outcomes.

## Conclusion

Having <23 teeth was significantly associated with the risk of requiring maintenance dialysis therapy in men aged <65 years. Although the results of this study suggest a significant association between tooth loss and ESKD, certain limitations must be considered. Future research should aim to address these limitations and further elucidate the complex relationship between oral health and renal function decline. Additionally, efforts to improve oral health among patients undergoing maintenance dialysis therapy through interdisciplinary collaboration between medical and dental professionals are warranted.
